# Genetic Ablation of Afadin Causes Mislocalization and Deformation of Paneth Cells in the Mouse Small Intestinal Epithelium

**DOI:** 10.1371/journal.pone.0110549

**Published:** 2014-10-21

**Authors:** Miki Tanaka-Okamoto, Yu Itoh, Jun Miyoshi, Akira Mizoguchi, Kiyohito Mizutani, Yoshimi Takai, Masahiro Inoue

**Affiliations:** 1 Department of Molecular Biology, Osaka Medical Center for Cancer and Cardiovascular Disease, Osaka, Japan; 2 Department of Biochemistry, Osaka Medical Center for Cancer and Cardiovascular Disease, Osaka, Japan; 3 Department of Neural Regeneration and Cell Communication, Mie University Graduate School of Medicine, Mie, Japan; 4 Division of Pathogenetic Signaling, Department of Biochemistry and Molecular Biology, Kobe University Graduate School of Medicine, Kobe, Hyogo, Japan; Institute of Molecular and Cell Biology (IMCB), Singapore

## Abstract

Afadin is an actin filament-binding protein that acts cooperatively in cell adhesion with the cell adhesion molecule nectin, and in directional cell movement with the small G protein Rap1 in a nectin-independent manner. We studied the role of afadin in the organization of the small intestinal epithelium using *afadin* conditional gene knockout (cKO) mice. Afadin was localized at adherens junctions of all types of epithelial cells throughout the crypt-villus axis. Paneth cells were localized at the base of the crypt in control mice, but not confined there, and migrated into the villi in *afadin*-cKO mice. The distribution of other types of epithelial cells did not change significantly in the mutant mice. The Paneth cells remaining in the crypt exhibited abnormal shapes, were buried between adjacent cells, and did not face the lumen. In these cells, the formation of adherens junctions and tight junctions was impaired. Rap1 and EphB3 were highly expressed in control Paneth cells but markedly down-regulated in the *afadin*-deficient Paneth cells. Taken together, the results indicate that afadin plays a role in the restricted localization of Paneth cells at the base of the crypt by maintaining their adhesion to adjacent crypt cells and inhibiting their movement toward the top of villi.

## Introduction

Paneth cells are one of the differentiated intestinal epithelial cells, which also include enterocytes, enteroendocrine cells, goblet cells, and tuft cells [1]. These cells are orderly lined up to form the villus-crypt axis. Paneth cells reside in the base of the crypt and play a role in antimicrobial defense [2]. Specifically, the Paneth cells reside next to the crypt base columnar (CBC) cells, which express Lgr5 [3]. CBC cells continuously divide and provide transiently amplifying (TA) cells, which undergo rapid cell division and subsequently migrate to the top of the villi after differentiation. In contrast, Paneth cells remain at the base of the crypt and mature by activation of the Wnt signal through the Wnt receptor Frizzled-5 [4]. The mechanism for confining Paneth cells to the base of the crypt has not been fully elucidated, but one proposed mechanism is repulsive interactions between EphB3-expressing crypt cells, such as Paneth and CBC cells, and ephrinB-expressing villus cells [5], [6]. A recent study revealed that Paneth cells also serve as niche cells for intestinal stem cells [3].

All types of epithelial cells in the small intestine attach to each other via an apical junctional complex of adherens junctions (AJs) and tight junctions (TJs) to form a monolayer sheet. Desmosomes are also involved in this attachment. AJs play a role in mechanically connecting adjacent cells to maintain tissue structure [7], [8]. In addition, AJs serve as a platform for intracellular signaling to regulate cell adhesion, polarization, movement, survival, proliferation, and differentiation [9]. TJs are localized on the most apical side of the cell–cell adhesion site, and AJs are formed on the basal side of TJs [10]. TJs are crucial for the barrier function that prevents the passage of soluble molecules through the gaps between cells and for the fence function that prevents the free diffusion of substances between apical and basolateral membrane domains [11]. AJs regulate the formation and maintenance of TJs [12].

The major cell adhesion molecules (CAMs) at AJs are cadherins and nectins [7], [9]. Cadherins are Ca^2+^-dependent CAMs that comprise a family consisting of over a hundred members [13]. Nectins are Ca^2+^-independent CAMs that comprise a family consisting of four members [14]. The major CAMs at TJs are junction adhesion molecules (JAMs), occludin, and claudins [10], [11], which are all Ca^2+^-independent. JAMs and claudins comprise a family consisting of four and approximately 24 members, respectively, whereas occludin does not comprise a family [11]. In the process of the formation of the apical junctional complex, nectins form cell–cell adhesions and then recruit cadherins to the nectin-based cell–cell adhesion site to form AJs. Subsequently, JAMs, occludin, and claudins are recruited to the apical side of AJs to form TJs [15].

Afadin was originally isolated as an actin filament-binding protein, which has a nucleotide sequence similar to that of *AF6* [16], [17]. Among the splicing isoforms of afadin, the longest isoform, l-afadin, hereafter referred to as afadin, is expressed ubiquitously [17]. Afadin is localized at AJs and binds to the cytoplasmic region of nectins, interacting directly with many proteins, such as Rap1 small G protein, α-catenin, p120^ctn^, PLEKHA7, ZO-1, ponsin, ADIP, LMO7, and drebrin [18], [19]. These interactions are involved in the formation of AJs with the cooperation of cadherins, and the formation of TJs with the cooperation of JAMs, occludin, and claudin. In addition to this role of afadin in the formation of the apical junctional complex, the protein is localized at the leading edge of moving cells and regulates directional cell movement [20]. This localization is regulated by binding to Rap1, which is activated in response to chemoattractants, such as platelet-derived growth factor (PDGF) and vascular endothelial growth factor, and required for directional cell movement, but not random cell movement [21], [22]. Activation of Rap1 by PDGF leads to the activation of the small G protein Rac1 and the inactivation of the small G protein RhoA [23]. Rap1, Rac1, and RhoA are cyclically activated and inactivated at the leading edge of directionally moving cells. Afadin plays a critical role in the cyclical activation and inactivation of these small G proteins.

Genetically modified mice with alterations in *afadin* have been generated and studied. Conventional *afadin*-knockout mice (KO) are embryonic lethal at E7.5 due to disorganization of the ectoderm, impaired migration of the mesoderm, and loss of somites and structures normally derived from both the ectoderm and mesoderm [24], [25]. Conditional *afadin*-KO mice (cKO) were subsequently generated, and studies using these mice have revealed the functions of afadin in various tissues. *Calcium-calmodulin-dependent kinase II-Cre-*mediated cerebrum-specific *afadin*-cKO causes an instability in synaptic junctions [26]. *Nestin-Cre*-mediated central nervous system-specific *afadin*-cKO mice develop severe hydrocephalus due to AJ dysfunction in the midbrain, and they died perinatally [27]. *Tie2-Cre*-mediated vessel endothelium-specific *afadin*-cKO mice exhibit impaired embryonic lymphangiogenesis and postnatal angiogenesis [28], [29]. Furthermore, characteristics of *Pax3-Cre*-mediated metanephric mesenchyme-specific *afadin-*cKO mice indicate that afadin is necessary for *de novo* lumen formation and elongation in the nephron [30]. Therefore, afadin plays roles *in vivo* in the formation and remodeling of organs and tissues by regulating the formation of cell–cell junctions and cell movement.

We previously generated *villin*-*Cre*-mediated intestinal epithelium-specific *afadin*-cKO mice [31]. These mutant mice exhibit increased paracellular permeability of intestinal epithelial cells and enhanced susceptibility to the tissue destruction induced by dextran sulfate sodium. Although the subcellular localization of nectin-2 and -3 is altered, the junctional architecture of most small intestinal epithelial cells is apparently preserved. In the present study, we show that afadin plays a role in the restricted localization of Paneth cells at the base of the crypt.

## Materials and Methods

### Mice

The targeting strategy for the disruption of *afadin* was described previously [26]. *Afadin*-floxed mice were mated with transgenic mice expressing Cre-recombinase under the control of the *villin* promoter (Jackson Laboratory, Bar Harbor, ME, USA) [32], and *afadin*-floxed/floxed mice with or without the *Cre* transgene were intercrossed. All of the results presented here were obtained from genetically inbred mice with the same mixture of the genetic background. First generation KO mice have a genetic background consisting of 129SV, C57BL/6, and DBA2 (50%, 25%, and 25%, respectively). They were further mated with C57BL/6 mice expressing Cre recombinase. The final genetic background of the mice conditionally lacking *afadin* was 129SV: C57BL/6: DBA2 = 37.5%: 43.75%: 18.75%. The animal care and experimental procedures in this study were specifically approved by the Institutional Animal Care and Use Committee (IACUC) of Osaka Medical Center for Cancer and Cardiovascular Diseases (Permit Number: 13060507) and carried out according to the institutional guidelines. All efforts were made to minimize suffering.

### Antibodies

Antibodies against the following proteins were purchased from commercial sources: afadin, chromogranin A, and DCAMKL (Dclk) (Abcam, Cambridge, UK); E-cadherin (R&D Systems, Minneapolis, MN, USA and BD Biosciences, San Jose, CA, USA); ZO-1 (Sanko-junyaku, Tokyo, Japan); Ki-67 (Novocastra Laboratories, Newcastle Upon Tyne, UK); lysozyme (DAKO, Glostrup, Denmark); cleaved caspase3 (Cell Signaling, Beverly, MA, USA); Rap1 (Millipore Corporation, Billerica, MA, USA); EphB3 (Abcam and R&D Systems); and EphB2 and ephrinB1 (R&D Systems). Alexa Fluor and horseradish peroxidase (HRP)-conjugated secondary antibodies were purchased from Millipore Corporation and Santa Cruz Biotechnology (Santa Cruz, CA, USA), respectively.

### Immunostaining and PAS staining

Mouse jejunum sections were fixed in 20% formalin neutral buffer solution, embedded in paraffin, and sectioned into 4-µm-thick sections. After deparaffinization, the sections were treated with an H_2_O_2_ solution and antigens retrieved by boiling with 10 mM sodium citrate buffer (pH 6.0). After blocking with 5% skimmed milk and 0.005% saponin in phosphate-buffered saline (PBS), the samples were incubated with primary antibodies at 4°C overnight and then with fluorescence or HRP-conjugated secondary antibodies for 30 minutes. For *Ulex europaeus* agglutinin 1 (UEA-1) staining, UEA-1 (Vector Laboratories, Burlingame, CA, USA) was used instead of the primary antibodies. For ephrinB1 staining, the sections were boiled in 20 mM Tris buffer (pH 9.0) for antigen retrieval and incubated in 1% BSA and 0.005% saponin in PBS for blocking. Chemiluminescence or fluorescence images were recorded on a charge-coupled device camera (Keyence) and a confocal microscope (Leica TCS SPE, Leica Microsystems, Wetzlar, Germany). PAS staining was performed based on standard protocol using periodic acid (Nacalai Tesque, Kyoto, Japan) and Cold Schiff’s reagent (Wako Pure Chemical Industries, Ltd., Osaka, Japan).

### BrdU labeling assay

Mice were intraperitoneally injected with 0.05 mg/g bromodeoxyuridine (BrdU) and sacrificed 2 hours later. Tissues were fixed in Carnoy’s solution, embedded in paraffin, and 4-µm sections stained with anti-BrdU antibody (DAKO).

### TUNEL staining

The intestinal sections were deparaffinized and subjected to TUNEL assay as described in the manufacturer’s instructions (Takara Bio Incorporation).

### Immunoprecipitation and Western blot

The colon cancer cell line Ls174T (DS Pharma Biomedical Co., Osaka, Japan) was cultured in MEM containing 1% NEAA, 2 mM L-glutamine, and 10% FBS and lysed in 50 mM Tris HCl (pH 7.5), 150 mM NaCl, 1 mM MgCl_2_, 1% Nonidet P-40, 1 mM EGTA, and 10% glycerol supplemented with 1 µg/ml aprotinin, 1 µg/ml leupeptin, 20 µg/ml phenylmethylsulfonyl fluoride, and phosphatase inhibitors. The lysate was clarified by centrifugation at 10,000×*g* for 10 minutes at 4°C. For immunoprecipitation, IgG or anti-afadin and EphB3 antibodies (ABcam; ab11338 and ab76885) were incubated with Dynabeads Protein G (Invitrogen) and added to 1 mg of pre-cleared lysate. The applied extracts were resolved in SDS polyacrylamide gels, electrophoretically transferred to a polyvinylidene difluoride membrane, and incubated with primary antibodies at 4°C overnight. The blots were subsequently incubated with HRP-conjugated secondary antibodies for 30 minutes and further treated with ECL Western Blotting Detection Reagents (GE Healthcare, Little Chalfont, UK).

### In situ hybridization

The jejuna obtained from control or *afadin*-cKO mice were fixed in 20% formalin neutral buffer solution, embedded in paraffin, and sectioned into 4-µm-thick sections. After deparaffinization, the sections were treated with the proteinase K solution, post-fixed, and treated in acetic anhydride solution. After prehybridization, the sections were incubated with RNA probe labeled with digoxigenin in the prehybridization solution (50% formamide, 5x SSPE, 5% SDS, and 1 mg/ml yeast tRNA) at 55°C overnight. Rinsed sections were blocked with 2% blocking reagent (Roche Diagnostics, Mannheim, Germany) and incubated with the anti-digoxigenin antibody conjugated to alkaline phosphatase (Roche Diagnostics) at 4°C overnight. Hybridized probe was visualized using BM purple (Roche Diagnostics). We used *Olfm4* RNA probe corresponding to the nucleotides, 218–851.

### Quantification of the staining images

Immunohistochemical staining intensity of Rap, EphB2, and EphB3 was quantified as follows. ROI (region of interest) was set on the region of crypt bottom as well as the non-crypt bottom as a internal control in each captured picture. The intensity was quantified by ImageJ, and the ratio of crypt/non-crypt was calculated. To measure the length of the villus-crypt axis and the numbers of BrdU-positive and apoptotic cells, vertical sections of crypt-villus axis were randomly observed in 6 to 10 fields per mouse and individual means compared.

### Statistical analysis

The student’s *t* test was used to evaluate the significance of differences between two mouse groups of different genotypes. P-values<0.05 were considered significant. All calculations were performed using Excel or Prism 6 (Graph Pad Software, La Jolla, CA).

## Results

### Mislocalization of Paneth cells in the small intestine of *afadin*-cKO mice

**Figure 1:**
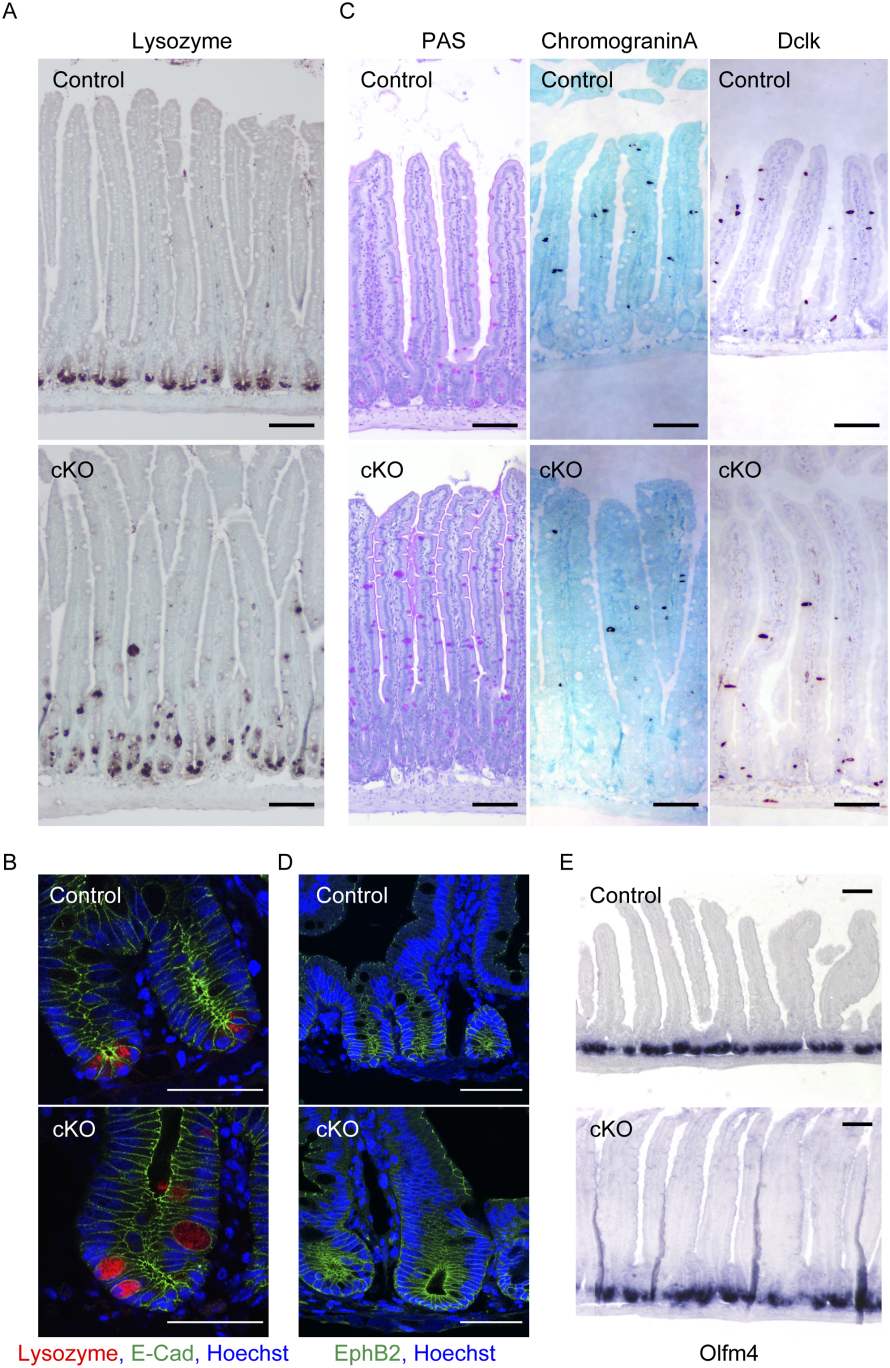
Mislocalization of Paneth cells in the small intestines of *afadin*-cKO mice. (**A–C**) Immunostaining of the small intestine of control and *afadin*-cKO mice with the indicated antibodies. (**A**) Paneth cell marker lysozyme. Scale bar = 100 µm. (**B**) Paneth cell marker lysozyme (red), adherens junction marker E-cadherin (green), and nuclear marker Hoechst33258 (blue). Scale bars = 50 µm. (**C**) Goblet cell marker PAS (left column), enteroendocrine marker chromograinin A (center column), and tuft cell marker Dclk1 (right column). Scale bars = 100 µm. (**D**) Immunostaining of the crypts in the small intestine of control and *afadin*-cKO mice with antibodies against EphB2 (green) and Hoechst33258 (blue). Scale bars = 50 µm. (**E**) Detection of *Olfm4* mRNA by *in situ* hybridization in the small intestine of the indicated mice. Scale bars = 100 µm.

First, we examined the role of afadin in the localization of enterocytes, goblet cells, enteroendocrine cells, Paneth cells, and tuft cells using the intestine-specific *afadin*-cKO mice generated previously [31]. Cells with large secretory granules containing lysozymes were identified as Paneth cells [33]. Goblet cells were detected by periodic acid–Schiff (PAS) staining. Enteroendocrine cells and tuft cells were shown by immunostaining with anti-chromogranin A or anti-Dclk antibodies, respectively [34], [35]. The immunochemical signal for lysozyme was restricted to the cells radially located in the base of the crypt of the small intestine of control mice, but it was scattered into the villus in the small intestine of *afadin*-cKO mice (Figure 1A). Furthermore, the lysozyme signal was apically located in the cytoplasm of control crypt cells. However, in *afadin*-cKO mice, the signal was diffusely distributed in the cytoplasm of the small intestine cells, and cells positive for lysozyme were round in shape and larger in size than control cells (Figure 1B). In contrast, the localization and morphologies of goblet cells, enteroendocrine cells, and tuft cells were indistinguishable between control and *afadin*-cKO mice (Figure 1C). CBC cells were recently shown to remain next to Paneth cells in the base of the crypt of the small intestine [3], [36]. Therefore, CBC cells were detected by immunostaining for EphB2 and performing *in situ* hybridization for *Olfm4*. The signals for EphB2 and *Olfm4* mRNA were confined to the base of the crypt in the small intestine of *afadin*-cKO mice, similar to the control crypts (Figure 1D, E). These results indicate that the genetic ablation of afadin causes the mislocation and deformation of Paneth cells, but not goblet cells, enteroendocrine cells, tuft cells, or CBC cells, in the small intestine.

### Disorganized cell adhesion in *afadin*-cKO Paneth cells

**Figure 2:**
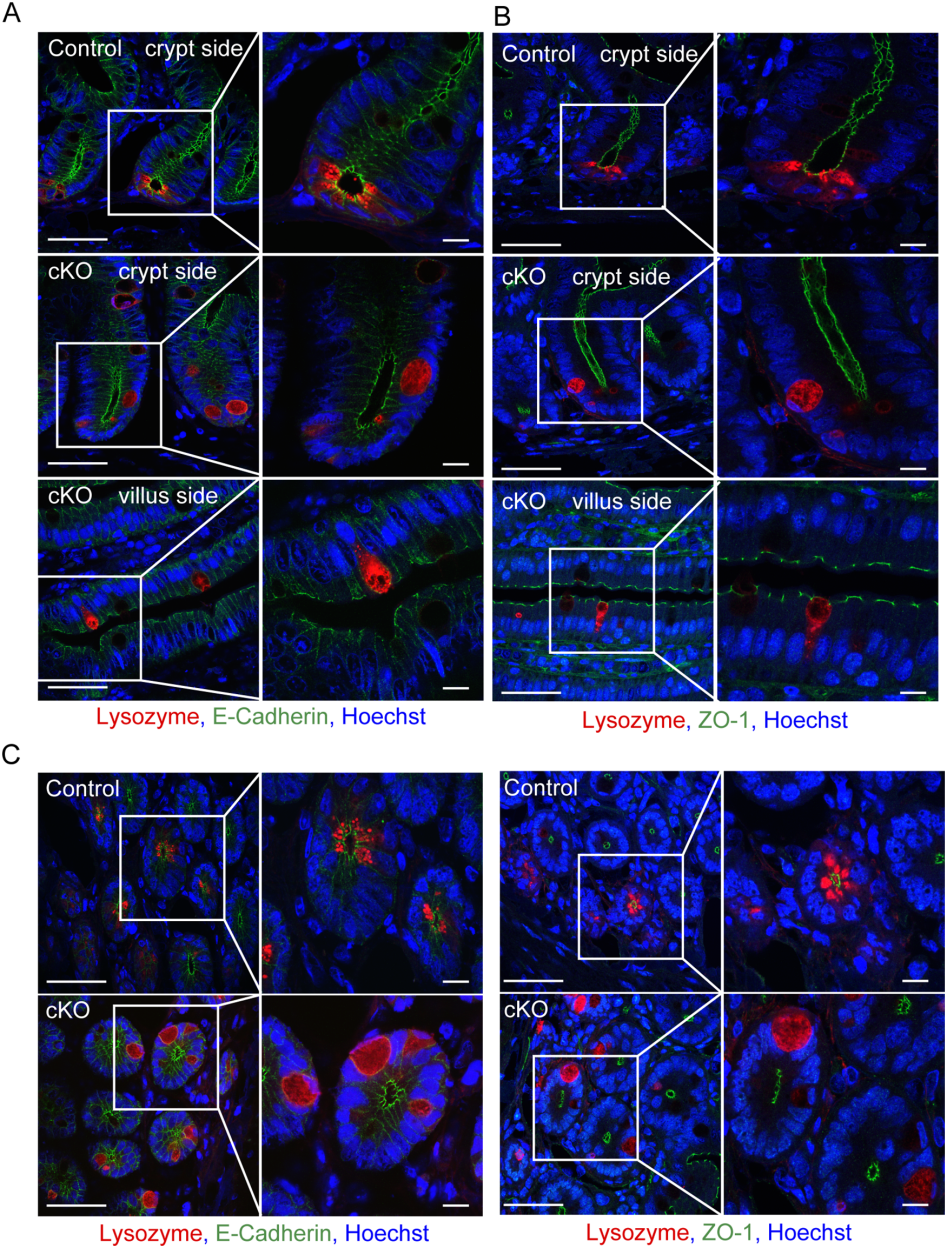
Impairment of the formation of AJs and TJs in *afadin*-cKO Paneth cells. (**A**) Immunostaining of the small intestine of control and *afadin*-cKO mice with antibodies against lysozyme (red), E-cadherin (green), and Hoechst33258 (blue). The upper two rows show the crypt side, and the bottom row shows a villus side of the crypt-villus axis from the indicated mice. Scale bars = 50 µm (left column), 10 µm (right column). (**B**) Immunostaining of the small intestine of control and *afadin*-cKO mice with antibodies against lysozyme (red), ZO-1 (green), and Hoechst33258 (blue). The upper two rows show the crypt side, and the bottom row shows a villus side of the crypt-villus axis from the indicated mice. Scale bars = 50 µm (left column), 10 µm (right column). (**C**) Immunostaining of the horizontal section of the crypt in the small intestine of control and *afadin*-cKO mice with antibodies against lysozyme (red), E-cadherin (green, left column), ZO-1 (green, right column), and Hoechst33258 (blue). Scale bars = 50 µm (left column), 10 µm (right column).

Afadin has been shown to play a role as a regulator for the formation of AJs and TJs in many types of epithelial cells, and that these cell–cell junctions prevent dissociation and movement of the attached cells [15]. Therefore, we examined whether AJs and TJs can form between adjacent cells in the small intestine of *afadin*-cKO mice. First, we confirmed the formation of AJs by staining E-cadherin, which was observed at the boundary between adjacent cells in the small intestine of control mice (Figure 2A, C, and Figure S1A, C). In the Paneth cells of *afadin*-cKO mice, the E-cadherin signal was not concentrated at the boundary between adjacent crypt cells, but a weak signal was distributed on the entire circumference like as other crypt cells (Figure 2A, C).

Next, we examined the formation of TJs by staining ZO-1, which is known to bind to the CAMs at TJs [10], [11]. ZO-1 was detected circumferentially at the most apical site between the adjacent intestinal epithelia, including Paneth cells, in the control small intestine. However, in *afadin*-cKO Paneth cells, the ZO-1 signal was hardly observed and lysozyme-positive cells were buried between adjacent crypt cells without the luminal membrane (Figure 2B, C, and Figure S1B, C). Some mislocated Paneth cells at the villus also lacked the ZO-1 signal, but the other mislocated Paneth cells at the villus had the apical membrane and E-cadherin and ZO-1 were detected at the boundary between Paneth cells and enterocytes (Figure 2A, B, and Figure S1D). These results indicate that afadin plays a role in the formation and/or maintenance of the AJs and TJs formed between Paneth cells and adjacent cells, at least in the crypt, which consistent with previous observations in MDCK cells [22] and suggest that afadin is involved in the localization of Paneth cells in the base of the crypt of the small intestine by regulating the formation and/or maintenance of the apical junctional complex.

### Down-regulation of Rap1 in *afadin*-cKO Paneth cells

**Figure 3:**
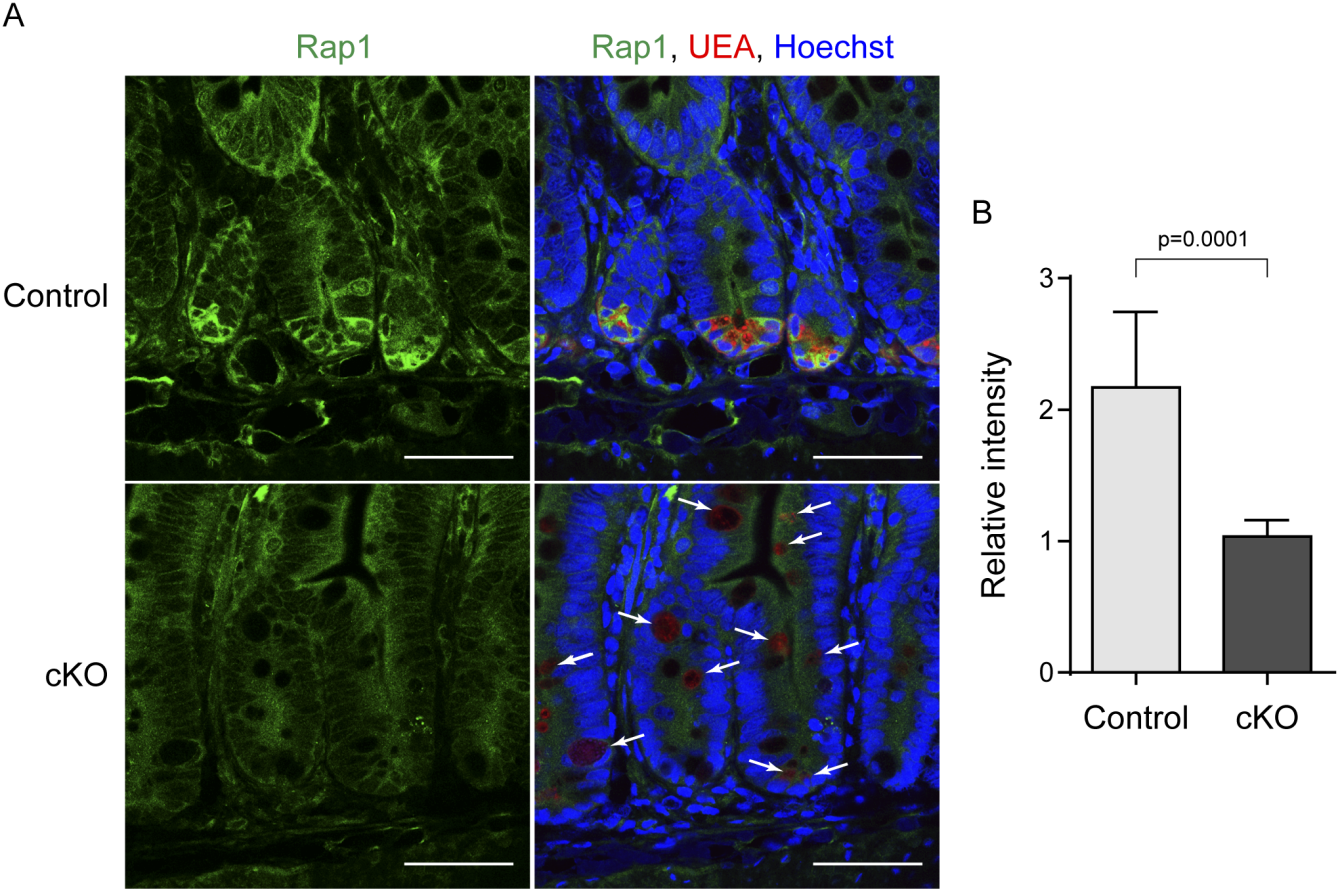
Down-regulation of Rap1 in *afadin*-cKO Paneth cells. (**A**) Immunostaining of the small intestine of control and *afadin*-cKO mice with antibody against Rap1 (green), the Paneth cell marker UEA-1 (red), and Hoechst33258 (blue). Arrows indicate low UEA cells. Scale bars = 50 µm. (**B**) Quantification of Rap signal. Relative intensity of Rap signal in crypt cells to that of non-crypt cells is shown. 37 crypts from 11 pictures (control) and 20 crypts from 7 pictures (*afadin*-cKO) were analyzed.

Afadin plays a role in directional cell movement by binding Rap1 [23], [37], [38], which is known to regulate cell motility through cytoskeletal remodeling [39]. Therefore, we investigated the expression of Rap1 in the small intestine. In the control crypt, the immunofluorescence signal for Rap1 was clearly observed in UEA-1-positive Paneth cells (Figure 3A). In contrast, the Rap1 signal was hardly observed in *afadin*-cKO crypt Paneth cells (Figure 3A, B). Similarly, the Rap1 signal decreased in the mislocated Paneth cells in the villus. Furthermore, the UEA-1 signal was down-regulated in mislocalized *afadin*-cKO Paneth cells, although the strong lysozyme signal was retained in the same cells (Figure 3A and Figure S2), possibly due to immature status of *afadin*-cKO Paneth cells [40]. These results indicate that the genetic ablation of afadin down-regulates Rap1 in Paneth cells and suggest that afadin is involved in the localization of Paneth cells in the base of the crypt of the small intestine by cooperatively regulating cell movement with Rap1.

### Down-regulation of EphB3 in *afadin*-cKO Paneth cells

**Figure 4:**
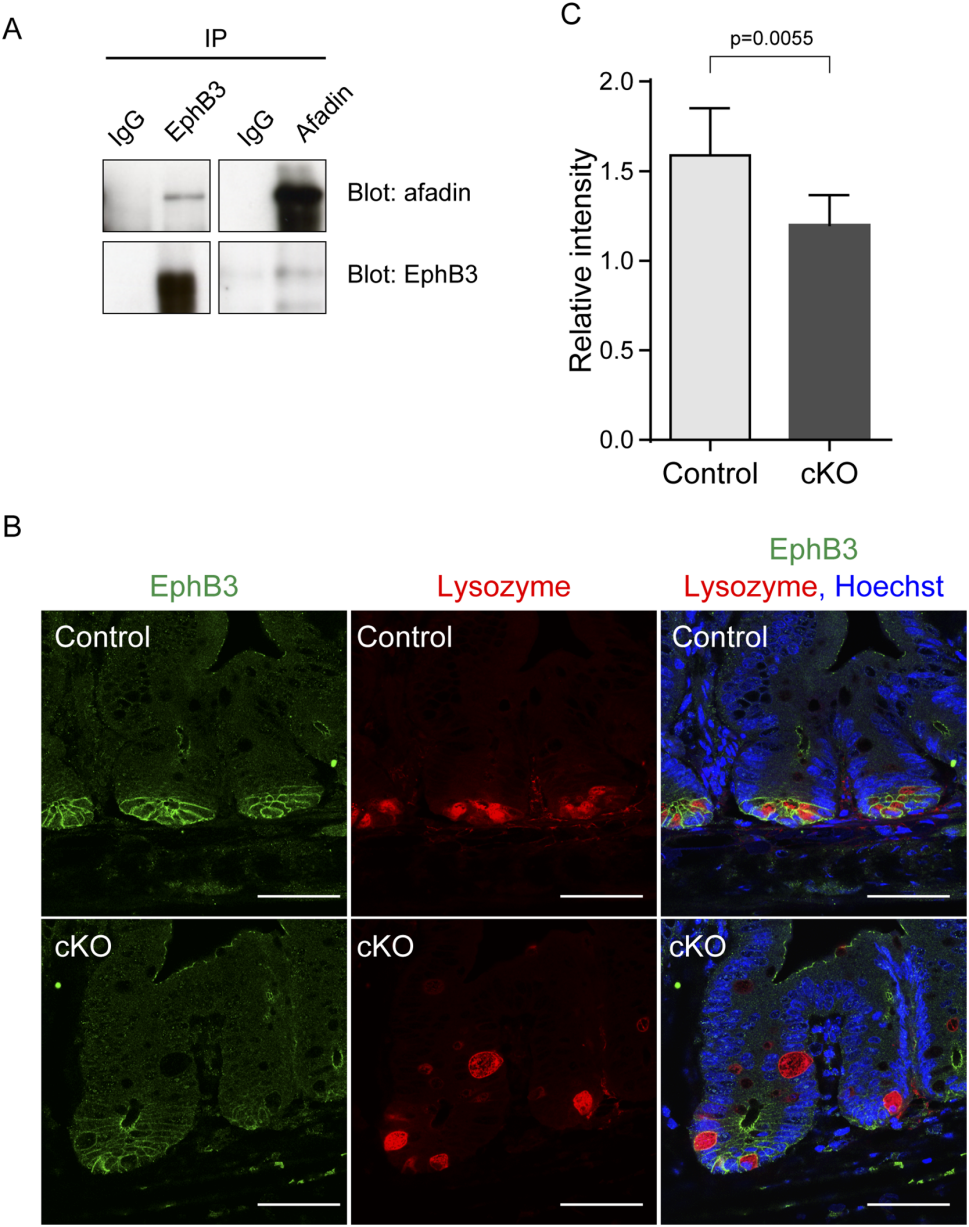
Interaction of afadin with EphB3 in the small intestine. (**A**) Western blotting of afadin and EphB3 using immunoprecipitates with the indicated antibodies from the Ls174T colon cancer cell line. (**B**) Immunostaining of the small intestine of control and *afadin*-cKO mice with antibodies against EphB3 (green), lysozyme (red), and Hoechst33258 (blue). Scale bars = 50 µm. (**C**) Relative intensity of EphB3 signal in crypt cells to that of non-crypt cells is shown. 18 crypts from 6 pictures (control) and 24 crypts from 8 pictures (*afadin*-cKO) were analyzed.

Paneth cells have been shown to mislocalized in *EphB3* and *ephrinB1* KO mice [5], [6]. In addition, afadin interacts with EphB3 [41]–[43]. Therefore, we examined whether EphB3 is involved in the mislocalization of Paneth cells in the small intestine of *afadin*-cKO mice. First, we confirmed the interaction of afadin with EphB3 using Ls174T colon cancer cells, which originate from a human colorectal tumor and expresses endogenous EphB3 and afadin (Figure S3). When EphB3 was immunoprecipitated from the cell lysate using the anti-EphB3 antibody, afadin co-immunoprecipitated with EphB3 (Figure 4A). Similarly, when afadin was immunoprecipitated from the cell lysate using the anti-afadin antibody, EphB3 co-immunoprecipitated with afadin. These results are consistent with earlier observations in neural tissues [41]–[43] and indicate that afadin interacts with EphB3.

Next, we assessed the localization of EphB3 in the small intestine of *afadin*-cKO mice. In control mice, the immunofluorescence signal for EphB3 was observed on the lateral membrane of Paneth cells and at the apical junctions of enterocytes (Figure 4B). The signal for afadin co-localized with that of EphB3 at the apical junctions of enterocytes along the crypt-villus axis (Figure S4A). In contrast, in the *afadin*-cKO crypt, EphB3 signal intensity decreased in the lateral membrane of crypt cells, though the EphB3 signal at the apical junctions of enterocytes remained unchanged (Figure 4B, C). By immunoblot analysis using extracts from whole small intestine or cultured crypts, the level of EphB3 protein was equally between both genotypes. The signal for EphB ligand ephrinB1 was detected along the lateral plasma membrane of the cells located on the villus side of the differentiated intestinal epithelial cells as described previously [5]. The ephrinB1 signal in *afadin*-cKO mice was similar to that of control mice (Figure S4B). These results indicate that afadin down-regulates the EphB3 receptor in the lateral membrane of crypt cells and suggest that afadin is involved in the confinement of Paneth cells in the base of the crypt of the small intestine by cooperatively regulating cell movement with EphB3.

### Increased length of the crypt-villus axis of the small intestine of *afadin*-cKO mice

**Figure 5:**
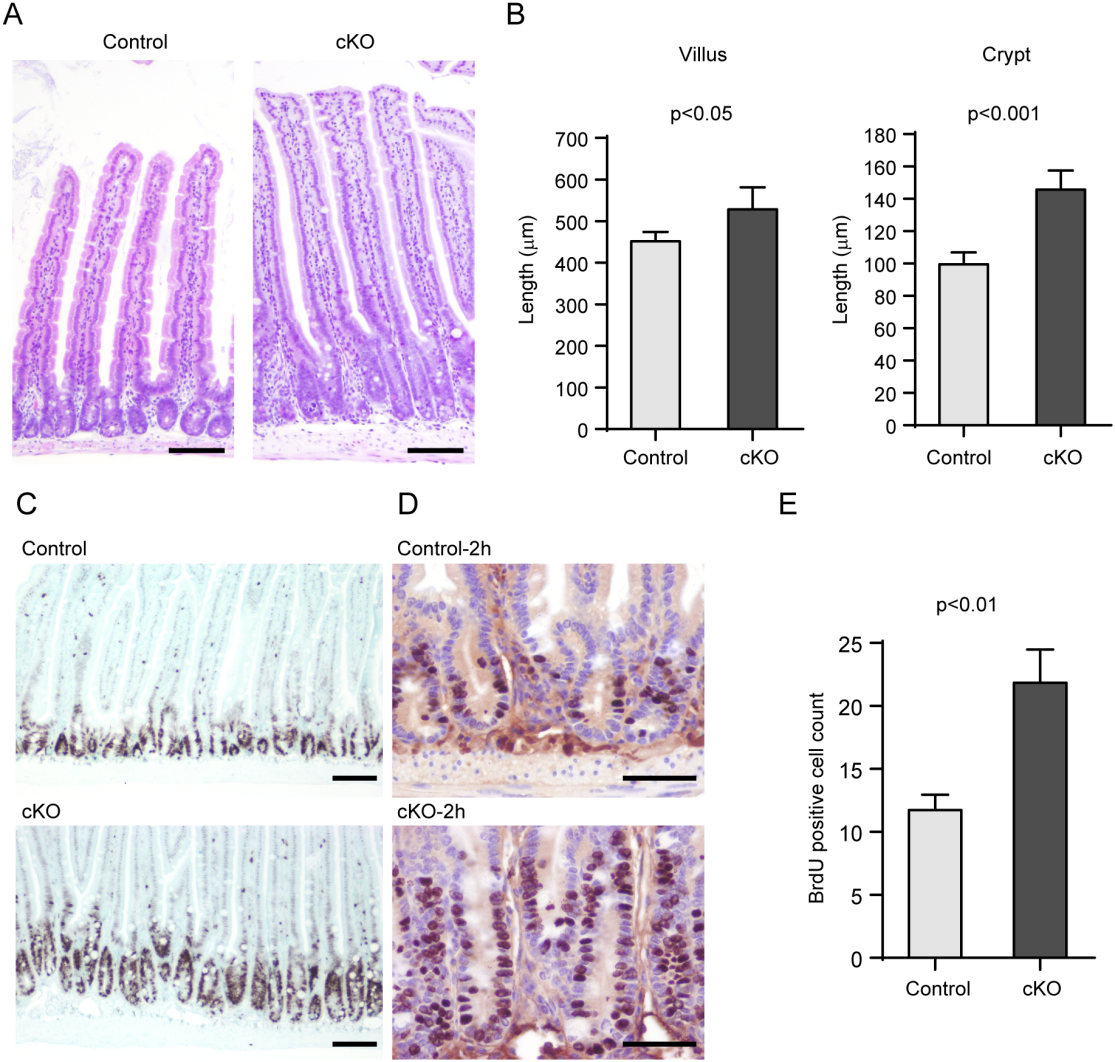
Increased length and enhanced proliferation of the crypt-villus axis of the small intestines of *afadin*-cKO mice. (**A**) Immunohistochemistry of the small intestine of control and *afadin*-cKO mice with hematoxylin and eosin. Scale bars = 100 µm. (**B**) Histogram showing the length of villi (left panel) and crypts (right panel), respectively (n = 6). (**C**) Immunohistochemistry of the small intestine of control and *afadin*-cKO mice with anti-Ki-67 antibody. Scale bars = 100 µm. (**D**) Immunohistochemistry of proliferating cells pulse-labeled with BrdU for 2 hours after peritoneal injection. Scale bars = 50 µm. (**E**) Histogram showing the number of BrdU-positive cells in each crypt of control and *afadin*-cKO mice. At least six crypts were counted in each mouse (n = 3).

In addition to the mislocation of Paneth cells in the small intestine of *afadin*-cKO mice, we noticed that the length of the crypt-villus axis was longer than in the small intestine of control mice (Figure 5A, B). Therefore, we examined whether this elongation is caused by increased cell proliferation and/or decreased apoptosis. Using Ki-67 staining, we evaluated whether the proliferation of CBC cells and TA cells was enhanced. The proliferating zone labeled with anti-Ki-67 antibody expanded in the small intestine of *afadin*-cKO mice compared to control mice (Figure 5C). The results were consistent with the analysis of BrdU incorporation (Figure 5D, E). Notably, many BrdU-positive cells were detected at the base of the crypt in the small intestine of *afadin*-cKO mice, but rarely in the small intestine of control mice. These results indicate that the genetic ablation of afadin causes increased proliferation of CBC cells and TA cells.

**Figure 6:**
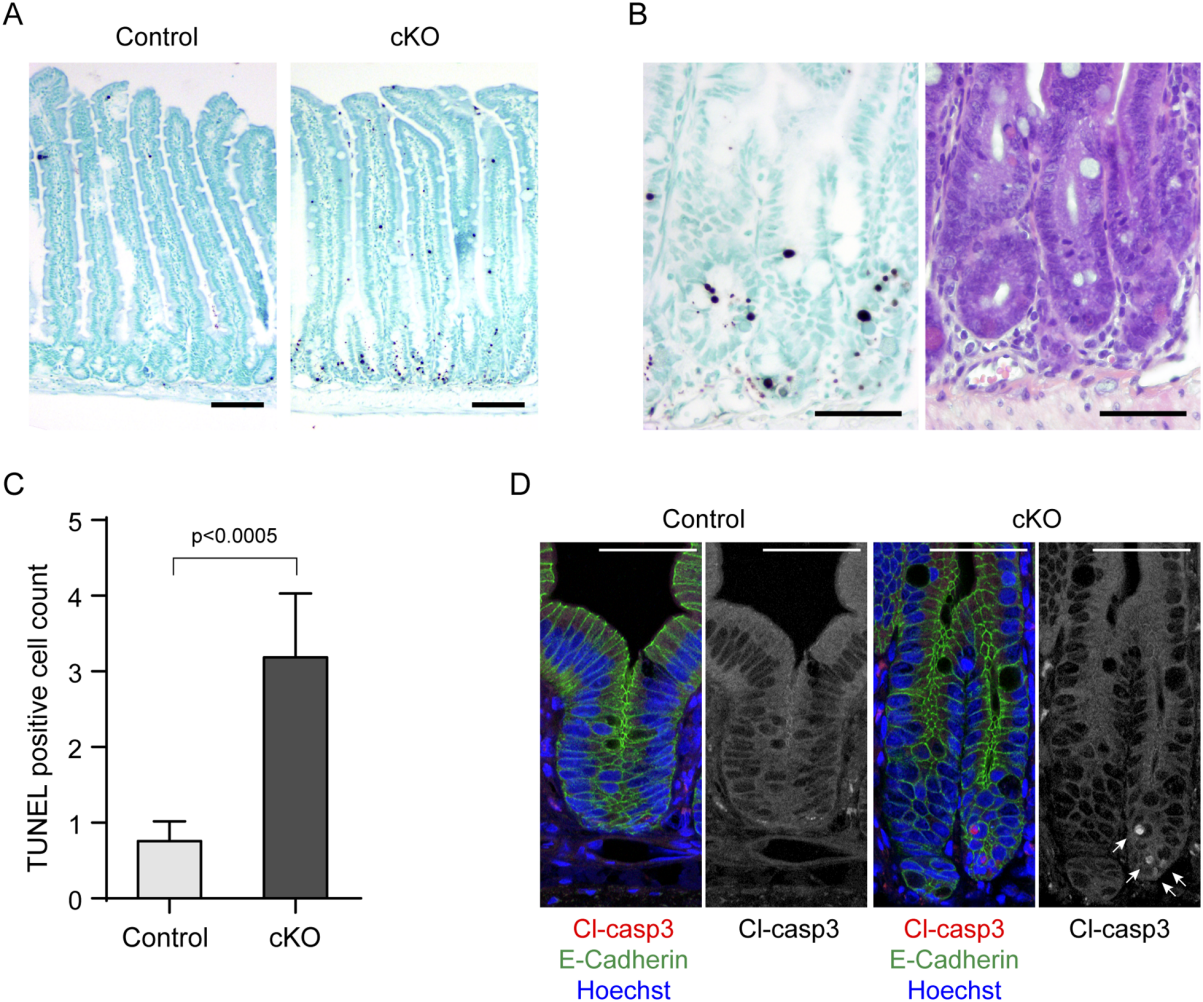
Enhanced death of the small intestinal epithelial cells in *afadin*-cKO mice. (**A**) TUNEL staining in the small intestine of control and *afadin*-cKO mice. Scale bars = 100 µm. (**B**) High magnification images of the TUNEL staining (left panel) and hematoxylin and eosin staining (right panel) of the serial section in the small intestine of *afadin*-cKO mouse. Scale bars = 50 µm. (**C**) Histogram showing the number of TUNEL-positive cells in each crypt-villus axis of the control and *afadin*-cKO mice. Ten axes were examined in each mouse (control, n = 5; *afadin*-cKO, n = 7). (**D**) Immunostaining of the small intestine of control and *afadin*-cKO mice with antibodies against E-cadherin (green), cleaved caspase-3 (cl-casp3: red), and Hoechst33258 (blue). Single channel images for cleaved caspase-3 are shown to the right of the merged pictures. Arrows indicate the apoptotic cells with cleaved caspase-3 signal. Scale bars = 50 µm.

Next, we examined whether cell death is increased in the small intestine of *afadin*-cKO mice using the TUNEL method. TUNEL-positive cells, which were rarely observed in the control mice, were mainly observed in the crypts of *afadin*-cKO mice (Figure 6A–C). In addition, cleaved caspase-3-positive cells, which were rarely observed in the control mice, were observed in the E-cadherin-labeled intestinal cells of the *afadin*-cKO mice (Figure 6D). These results indicate that the loss of afadin causes increased apoptosis of the intestinal epithelial cells, although we did not identify the cell type that died. Taken together, the results that the increased length of the crypt-villus axis in *afadin*-cKO mice is caused by a greater increase in the proliferation of CBC cells than the apoptosis of intestinal epithelial cells.

## Discussion

Here we showed that Paneth cells, but not goblet cells, enteroendocrine cells, tuft cells, or CBC cells, are mislocalized in the small intestine of *afadin*-cKO mice. *Afadin*-deficient Paneth cells exhibited a round morphology without the AJ and TJ structures at the base of the crypt. Furthermore, the Rap1 and EphB3 proteins were down-regulated in the *afadin*-cKO Paneth cells.

Radially arranged Paneth cells and CBC cells form the lumen at the base of the crypt in the small intestine. We found that the *afadin*-deficient Paneth cells remaining in the base of the crypt exhibit characteristic phenotypes: their shape became round, their size became large, they did not face the lumen, and they lost TJs and AJs. The mislocalization of Paneth cells has been observed in the small intestine of *E-cadherin*-deficient mice, as well as the polarity-related protein *Kruppel-like factor 4-* or *Cdc42*-deficient mice [44]–[47]. Taken together with these earlier observations, the present results suggest that afadin-dependent cell adhesion plays a critical role in confining Paneth cells in the base of the crypt. Loss of the apical junctions in the small intestine of *afadin*-cKO mice was restricted to Paneth cells while other types of the intestinal epithelial cells retained the apical junctions [31]. At the base of the crypt, Paneth cells and CBC cells heterotypically contact each other and are arranged in an orderly manner [3]. As CBC cells proliferate, Paneth cells must dynamically remodel the adhesion structure to maintain the regular pattern and reside in the base of the crypt. Thus, afadin might be required for the initiation of cell adhesion or cytoskeletal remodeling, although afadin might not be necessary for maintaining cell adhesion after it is established.

Rap1 has been shown to regulate a variety of cellular functions, such as cell survival, proliferation, differentiation, adhesion, polarization, and movement, through its downstream effectors [48]–[50]. Afadin is one of the downstream effectors of Rap1 and regulates cell adhesion, polarization, and movement in cooperation with Rap1 [23], [37], [39], [51], [52]. In addition, afadin regulates the activation of Rap1 by inhibiting its inactivation by the Rap1 GAP SPA-1; thus, afadin plays a role in regulating a variety of Rap1-mediated cellular functions [23]. We showed that Rap1 is highly expressed in Paneth cells compared to other types of cells in the small intestine, but it was not observed in *afadin*-deficient Paneth cells. Rap1 may be down-regulated in Paneth cells in the absence of afadin, although the mechanism remains unknown. Together with earlier observations, the present results indicate that afadin plays a role in maintaining a sufficient level of Rap1 protein in Paneth cells to exert its activity through afadin and regulate the localization of Paneth cells in the base of the crypt in cooperation with Rap1.

In the small intestinal epithelium, EphB3 is localized in the cells present at the base of the crypt, such as Paneth cells and CBC cells, and its ligand ephrinB is expressed in the cells located on the villus side of the differentiated intestinal epithelial cells [5]. Paneth cells are mislocated in *EphB3-* and *ephrinB1-*KO mice, suggesting that Paneth cells remain in the base of the crypt through the repulsive interaction of EphB3 and ephrinB [5], [6]. The PDZ domain of afadin interacts with the C-terminal domain of EphB receptors, including EphB2, -3, -5, and -6 and EphA7 [41]–[43]. We confirmed here for the first time that afadin interacts with EphB3 in the colon cancer cell line Ls174T. We then showed that EphB3 localizes not only along the lateral plasma membrane of Paneth cells, including the apical junctions, but also at the apical junctions of enterocytes along the crypt-villus axis. In the small intestine of *afadin*-cKO mice, EphB3 was down-regulated in Paneth cells at the base of the crypt, but not at the apical junctions of enterocytes. We showed that afadin colocalized with EphB3 at the apical junctions, but not at the lateral plasma membrane. Afadin is strictly localized at AJs in many types of epithelial cells, including small intestinal enterocytes [17]. In addition, afadin colocalizes with EphB3 in transfected human embryonic kidney 293T cells [42]. Taken together, the results indicate that afadin interacts with EphB3 at AJs in Paneth cells and enterocytes. As AJs were disrupted and EphB3 down-regulated in *afadin*-deficient Paneth cells at the base of the crypt, afadin-dependent localization of EphB3 at the AJs of Paneth cells may be required for the confinement of these cells at the base of the crypt. The exact mechanism for the down-regulation of EphB3 at the lateral plasma membrane, including the apical junctions, of Paneth cells but not at the apical junctions of enterocytes is unknown. However, EphB3 interacting with afadin at the apical junctions of Paneth cells and enterocytes may be more sensitive to the down-regulation than EphB3 that does not interact with afadin at the lateral plasma membrane lacking apical junctions. In contrast to EphB3, EphB2 signal was maintained in *afadin*-cKO crypts, probably because change in *afadin*-cKO small intestine was limited in Paneth cells, and CBC cells remained in the crypt which express only EphB2 but not EphB3.

We noted that the length of the crypt-villus axis of the small intestine of *afadin*-cKO mice was longer than that of the control mice. Although both the proliferation of CBC cells and the apoptosis of unidentified epithelial cells increased, the length of the crypt-villus axis was elongated in the small intestine of *afadin*-cKO mice. The extent of cell proliferation is likely greater than that of apoptosis. In the crypts of *Sox9* cKO mice, Paneth cells and CBC cells disappear and proliferative cells, presumably TA cells, increase [3], [53], [54]. In addition, the ablation of Paneth cells by diphtheria toxin results in the disappearance of CBC cells and an increase in proliferative cells, presumably TA cells [3], [55]. In contrast, in *afadin*-cKO crypts, the CBC cells seemed to remain intact. Mislocalization of Paneth cells alone may not be enough to eliminate CBC cells, and direct and persistent contact between CBC cells and Paneth cells may not be critical to maintaining CBC cells. Alternatively, afadin in CBC cells may play a role in eliminating CBC cells in the absence of Paneth cells. Further studies are necessary to understand the exact role and mode of action of afadin in the regulation of cell proliferation and apoptosis.

## Supporting Information

Figure S1 **E-cadherin and ZO-1 staining in controls and** ***afadin*** **-cKO mice. (A–C)** Single channel images of E-cadherin **(A, C)**, ZO-1 **(B, C)** in vertical sections **(A, B)** and horizontal sections **(C)**, corresponding to Figure 2. **(D)** Immunostaining images of the villus side in control small intestine with antibodies against lysozyme (red), E-cadherin (green, left column), ZO-1 (green, right column), and Hoechst33258 (blue) area shown as controls of Figure 2A and 2B. Scale bars = 50 µm (left column), 10 µm (right column).
(TIF)

Figure S2 **Down-regulation of Paneth cell markers in the small intestines of** ***afadin*** **-cKO mice.** Immunostaining of Paneth cells by two different Paneth cell markers: UEA-1 (red) and lysozyme (green). Note the decrease in UEA levels in the (arrows). Scale bars = 50 µm.
(TIF)

Figure S3 **Interaction of afadin with EphB3.** Western blotting of afadin and EphB3 in Figure 4A is shown with whole cell lysate of the small intestine, the colon, and Ls174T cells.
(TIF)

Figure S4 **Localization of EphB3, ephrinB1, and afadin in the small intestines of control and** ***afadin*** **-cKO mice.** (**A**) Immunostaining of the small intestines with EphB3 (red), afadin (green), and Hoechst33258 (blue). Scale bars = 50 µm. (**B**) Immunostaining of the small intestines with ephrinB1 (green), lysozyme (red), and Hoechst33258 (blue). Scale bars = 50 µm.
(TIF)
