# Supplementary figures and images for: Genetic Ablation of Afadin Causes Mislocalization and Deformation of Paneth Cells in the Mouse Small Intestinal Epithelium
Source: PLoS One. 2014 Oct 21;9(10):e110549. doi: 10.1371/journal.pone.0110549 (PMC4204899; doi:10.1371/journal.pone.0110549)

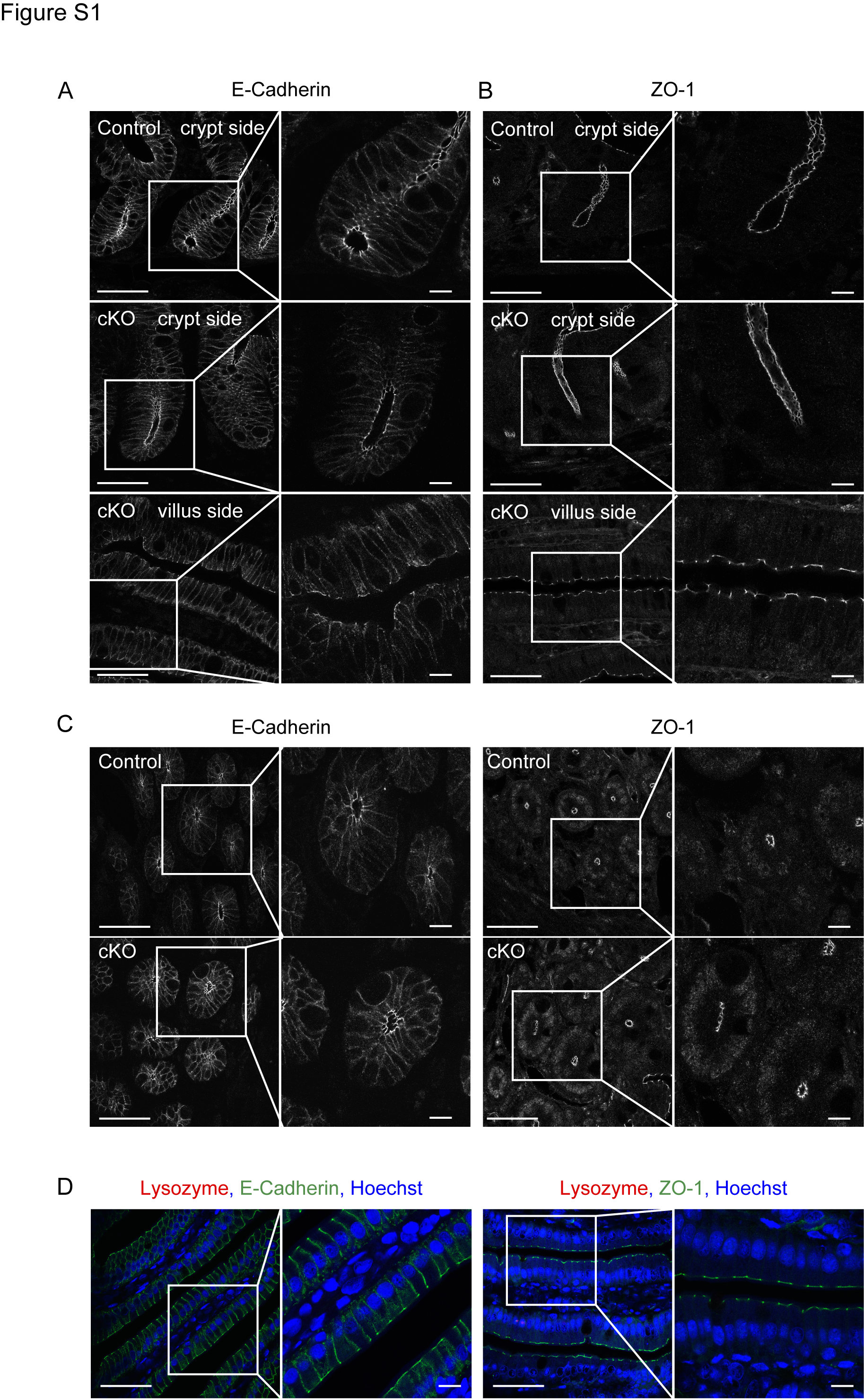

Supplement: Figure S1 — E-cadherin and ZO-1 staining in controls and afadin -cKO mice. (A–C) Single channel images of E-cadherin (A, C), ZO-1 (B, C) in vertical sections (A, B) and horizontal sections (C), corresponding to Figure 2. (D) Immunostaining images of the villus side in control small intestine with antibodies against lysozyme (red), E-cadherin (green, left column), ZO-1 (green, right column), and Hoechst33258 (blue) area shown as controls of Figure 2A and 2B. Scale bars = 50 µm (left column), 10 µm (right column). (TIF) [file pone.0110549.s001.tif]

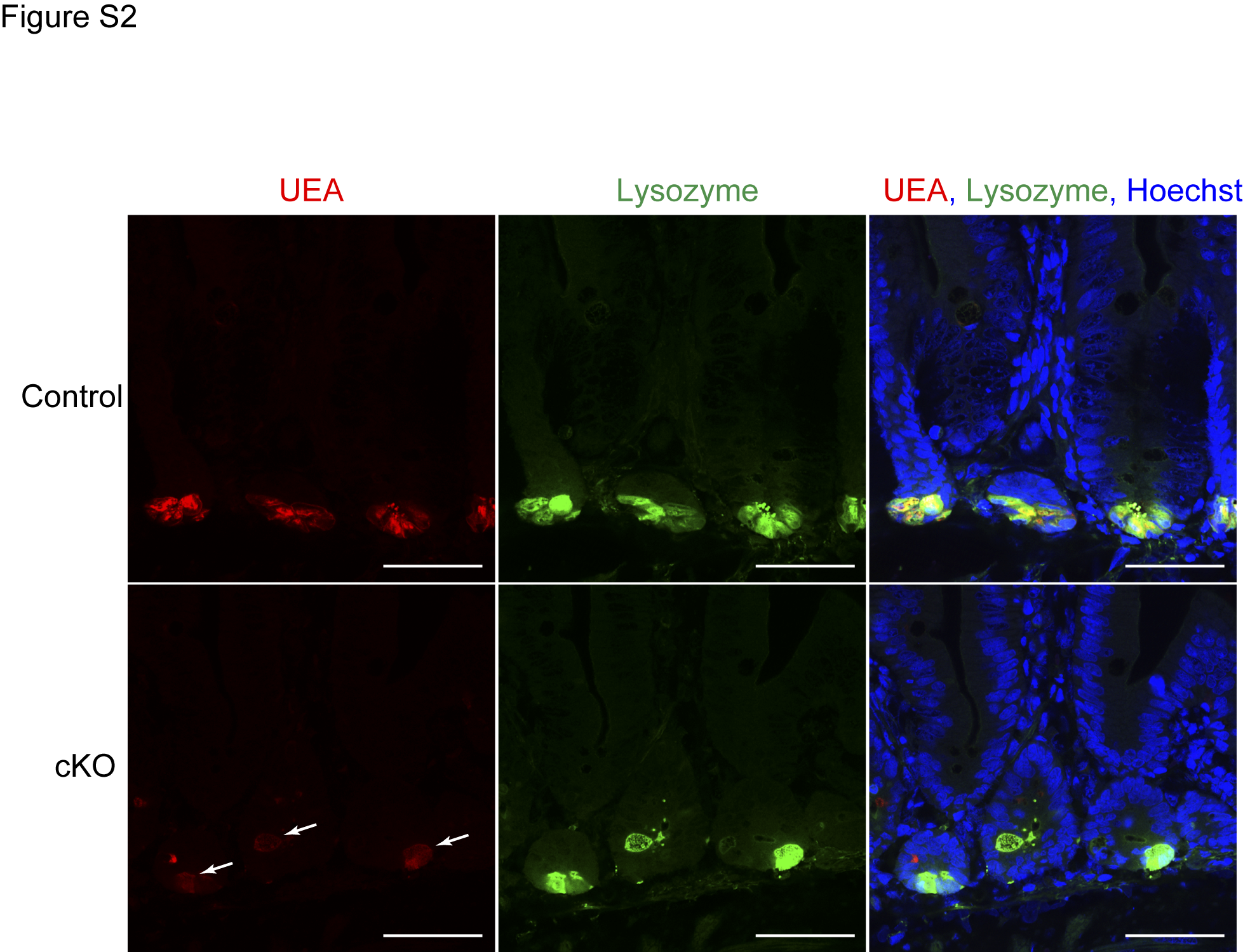

Supplement: Figure S2 — Down-regulation of Paneth cell markers in the small intestines of afadin -cKO mice. Immunostaining of Paneth cells by two different Paneth cell markers: UEA-1 (red) and lysozyme (green). Note the decrease in UEA levels in the (arrows). Scale bars = 50 µm. (TIF) [file pone.0110549.s002.tif]

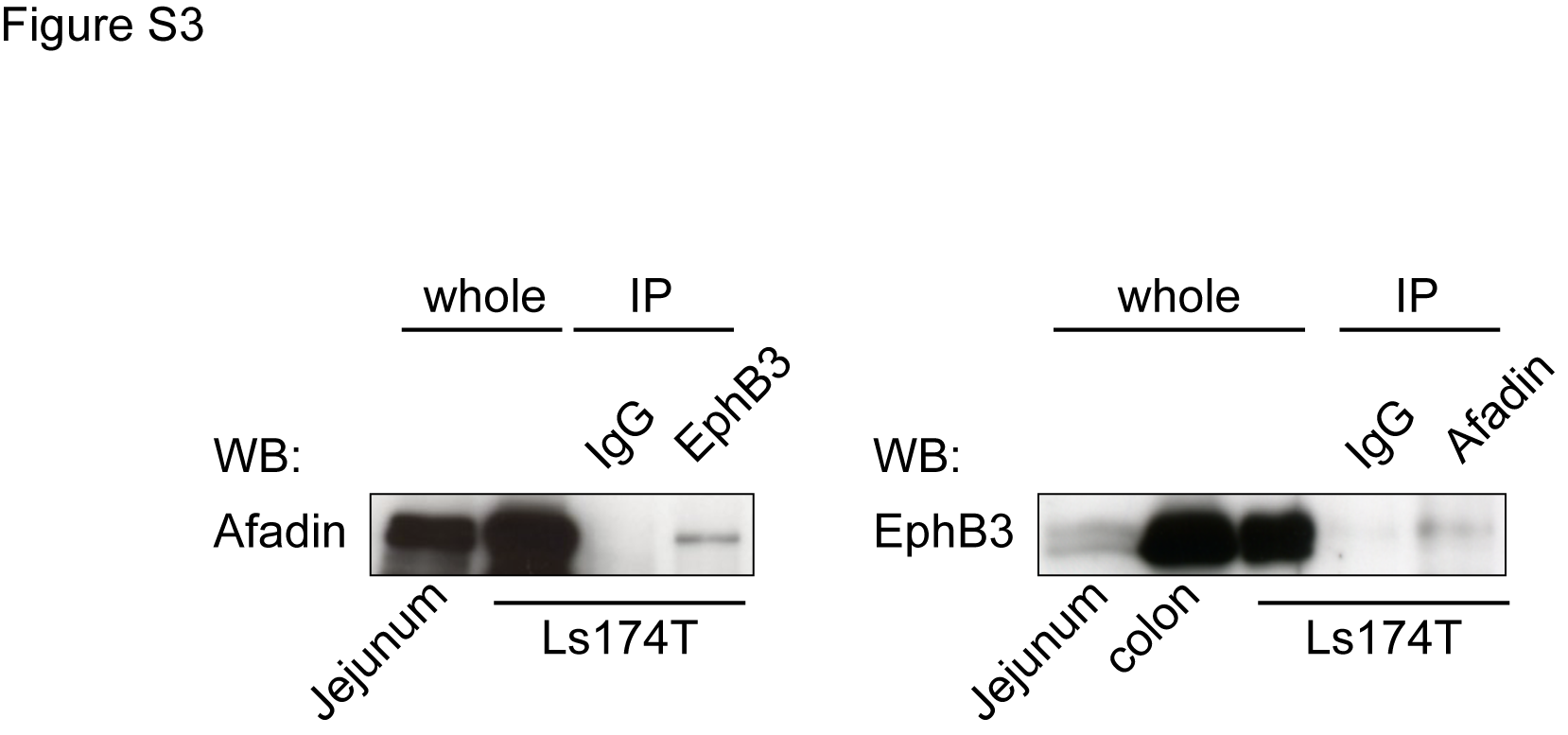

Supplement: Figure S3 — Interaction of afadin with EphB3. Western blotting of afadin and EphB3 in Figure 4A is shown with whole cell lysate of the small intestine, the colon, and Ls174T cells. (TIF) [file pone.0110549.s003.tif]

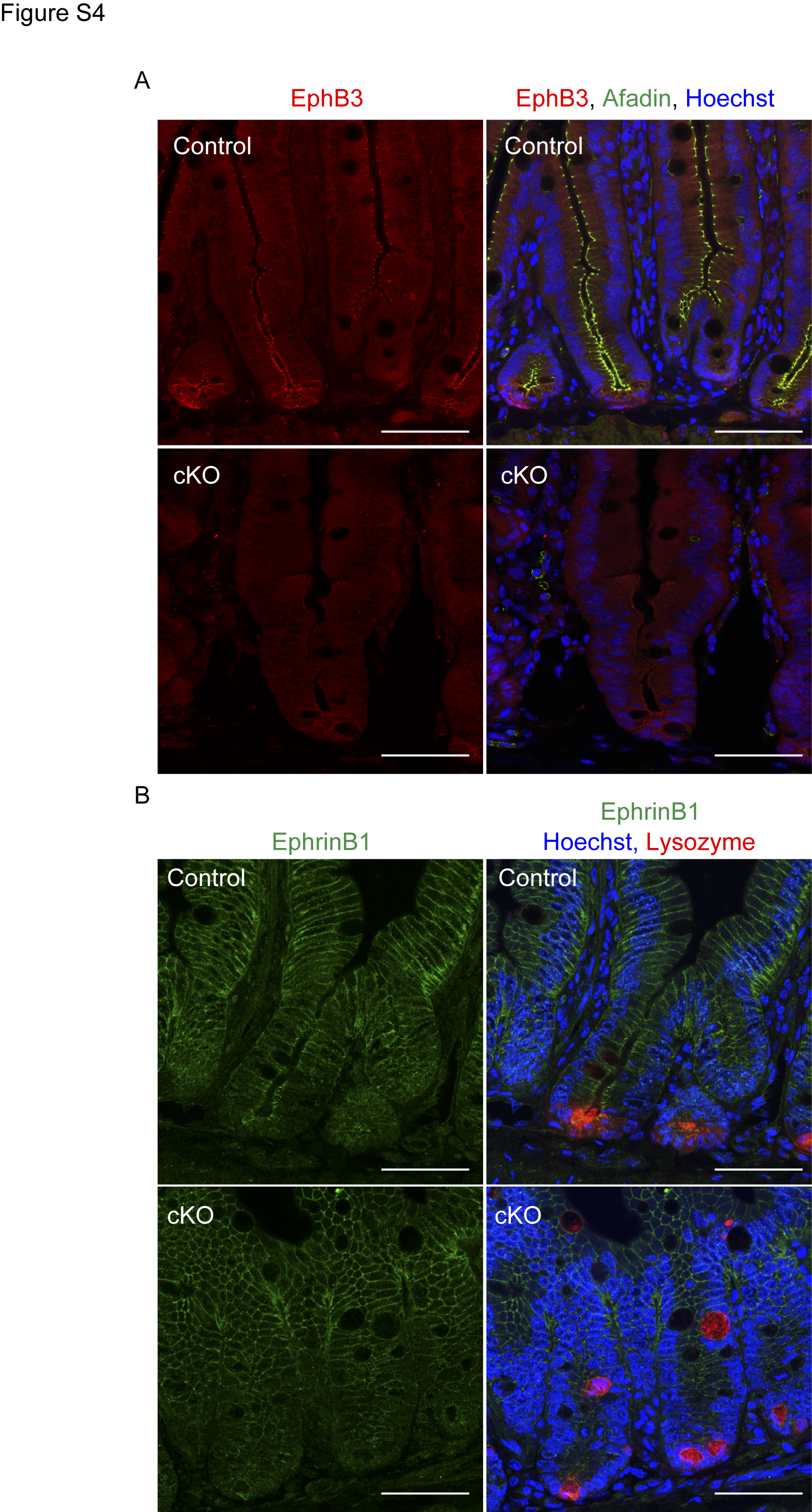

Supplement: Figure S4 — Localization of EphB3, ephrinB1, and afadin in the small intestines of control and afadin -cKO mice. (A) Immunostaining of the small intestines with EphB3 (red), afadin (green), and Hoechst33258 (blue). Scale bars = 50 µm. (B) Immunostaining of the small intestines with ephrinB1 (green), lysozyme (red), and Hoechst33258 (blue). Scale bars = 50 µm. (TIF) [file pone.0110549.s004.tif]
